# Exploring variability in basal ganglia connectivity with functional MRI in healthy aging

**DOI:** 10.1007/s11682-018-9824-1

**Published:** 2018-02-13

**Authors:** Ludovica Griffanti, Philipp Stratmann, Michal Rolinski, Nicola Filippini, Enikő Zsoldos, Abda Mahmood, Giovanna Zamboni, Gwenaëlle Douaud, Johannes C. Klein, Mika Kivimäki, Archana Singh-Manoux, Michele T. Hu, Klaus P. Ebmeier, Clare E. Mackay

**Affiliations:** 1 Centre for the functional MRI of the Brain (FMRIB), Wellcome Centre for Integrative Neuroimaging, Nuffield Department of Clinical Neurosciences, University of Oxford, Oxford, UK; 2 Oxford Parkinson’s Disease Centre (OPDC), Oxford, UK; 3 Department of Psychiatry, University of Oxford, Oxford, UK; 4 Department of Informatics, Germany and Institute of Robotics and Mechatronics, German Aerospace Center (DLR), Technical University of Munich, Wessling, Germany; 5 Nuffield Department of Clinical Neurosciences, University of Oxford, Oxford, UK; 6 Institute of Clinical Neurosciences, University of Bristol, Bristol, UK; 7 Department of Epidemiology and Public Health, University College London, London, UK; 8 INSERM, U 1018, Hôpital Paul-Brousse, Villejuif, France; 9 Oxford Health NHS Foundation Trust, Oxford, UK; 10 Oxford Centre for Human Brain Activity, Wellcome Centre for Integrative Neuroimaging, Department of Psychiatry, University of Oxford, Warneford Hospital, Oxford, OX3 7JX UK

**Keywords:** Functional connectivity, Basal ganglia, Resting state fMRI, Healthy aging, Dopamine, Parkinson’s disease

## Abstract

Changes in functional connectivity (FC) measured using resting state fMRI within the basal ganglia network (BGN) have been observed in pathologies with altered neurotransmitter systems and conditions involving motor control and dopaminergic processes. However, less is known about non-disease factors affecting FC in the BGN. The aim of this study was to examine associations of FC within the BGN with dopaminergic processes in healthy older adults. We explored the relationship between FC in the BGN and variables related to demographics, impulsive behavior, self-paced tasks, mood, and motor correlates in 486 participants in the Whitehall-II imaging sub-study using both region-of-interest- and voxel-based approaches. Age was the only correlate of FC in the BGN that was consistently significant with both analyses. The observed adverse effect of aging on FC may relate to alterations of the dopaminergic system, but no unique dopamine-related function seemed to have a link with FC beyond those detectable in and linearly correlated with healthy aging.

## Introduction

It is well established that in absence of specific tasks, the brain is organised into largely independent resting state networks (RSN) (Smith et al. 2009), detectable using resting state fMRI (rfMRI). The basal ganglia resting state network (BGN), although often not reported as one of the main RSNs, is identifiable, reproducible (across subjects, resting state conditions and imaging parameters), and corresponds with the motor control circuit, opening a new way to investigate the functional connectivity of the basal ganglia (Robinson et al. 2009; Di Martino et al. 2008).

Aberrant functional connectivity (FC) within the BGN has been observed in pathologies with altered neurotransmitter systems and conditions involving motor control and dopaminergic processes in general. In particular, reduced FC has been observed in patients with early Parkinson’s disease (PD) relative to healthy controls (HC) (Szewczyk-Krolikowski et al. 2014; Tan et al. 2015). This alteration appears not to be related to Alzheimer’s disease (Rolinski et al. 2015) but is present in individuals at risk of developing PD (patients with REM sleep behavior disorder) (Rolinski et al. 2016), suggesting that FC determined by rfMRI may be a promising biomarker for PD. Changes in FC of the BGN have also been observed in other dopamine-related conditions such as depression (Hwang et al. 2016), schizophrenia (Duan et al. 2015), and impulsive behaviors (Schmidt et al. 2015).

To date the factors that affect FC measured in the BGN remain unclear. Given the potential of rfMRI to detect changes due to altered neurotransmitter systems, we sought to explore whether a link with dopaminergic processes that is detectable with rfMRI is present in healthy older subjects. The hypothesis that changes in the nigrostriatal dopaminergic system already occur in normal aging is supported by previous PET and SPECT studies (Reeves et al. 2002). Therefore, a better characterization of variability of FC in healthy subjects might provide insight into the biological basis of the difference in FC found in pathological conditions. Moreover, identifying factors that account for this variability can also help to increase the specificity of potential biomarkers based on resting state FC. Statistically controlling for factors that are related to normal aging should increase power to detect disease-specific changes.

To this aim we used data from 486 healthy individuals aged 60 to 82 years participating in the Whitehall II imaging (WHII) sub-study (Filippini et al. 2014), which included brain MRI, demographic and health data, and tests of cognitive and motor performance. These data provided sufficient statistical power to investigate the relationship between FC in the BGN and a wide range of variables related to demographics, impulsive behavior, self-paced tasks, mood and motor correlates. To cross-validate findings, we used both a region-of-interest (ROI) and a voxel-wise approach.

## Methods

Brain MRI data from 486 participants in the WHII sub-study were analysed (age 69.45 ± 5.23 years, range 60–82 years, M/F = 388/98, education 14.00 ± 3.12 years). Details of the study, the MRI acquisition protocol, and pre-processing steps are described elsewhere (Filippini et al. 2014). Briefly, rfMRI data pre-processing included motion correction, brain extraction, high-pass temporal filtering (cut-off 100 s), field-map correction, artefact removal (FSL-FIX), spatial smoothing (FWHM 6 mm), registration to the individual structural (high-resolution T1-weighted) scans and to standard space (FNIRT, optimized using BBR approach). The template of RSNs used in this study was derived from a separate set of 45 age-matched elderly healthy controls, using group ICA with dimensionality d = 50, (which enabled reliable extraction of the BGN) as described by (Griffanti et al. 2016). Dual-regression was used to generate subject-specific maps of parameter estimates (PE) for the 50 components (29 RSNs and 21 artefacts) from pre-processed rfMRI data of the 486 subjects. Subject-specific BGN maps entered subsequent FC analyses. Structural T1 images of the individual subjects were segmented using FIRST (Patenaude et al. 2011) and the volume of BG structures (caudate nucleus, putamen and globus pallidus) was calculated to be included as covariate.

We tested the potential links between FC in the BGN and different domains using both a region-of-interest (ROI) and voxel-wise approach. Characteristics of the subjects are summarised in Table 1 (see (Filippini et al. 2014) for more details about the tests). The variables included in each domain and the tests performed are summarised in Table 2.

**Table 1:** Characteristics of the subjects

Variable	N (subjects with available data)	Mean	Std. deviation
Demographics			
Age (years)	486	69.45	5.23
Handedness (scale from − 24 (left) to 24 = (right) handed)	482	17.12	12.79
Sex (M/F)	486	388 / 98	
Impulsive behavior			
Current smoking (non-smoker / occasional / smoker)	486	465/3/18	
Alcohol consumption (units per week)	477	15.34	15.06
Body mass index	486	26.19	4.15
Self-paced tasks			
Letter fluency (average N)	486	15.69	4.57
Categorical fluency (average N)	486	22.09	5.68
Mood			
CES-D	485	5.22	6.21
Motor performance and sleep			
CANTAB RTI MT (average simple Movement Time, msec)	480	273.52	88.42
CANTAB RTI RT (average simple Reaction Time, msec)	480	317.07	79.21
Pegboard assembly task (average N)	272	26.06	6.13
Pegboard both hands (average N)	272	9.80	1.78
Pegboard left hand (average N)	272	12.11	2.07
Pegboard right hand (average N)	275	12.29	2.00
PSQI 11c (0/1/2/3)§	478	390/48/23/17	

Legend: CES-D = Centre for Epidemiological Studies Depression Scale; CANTAB RTI = Cambridge Neuropsychological Test Automated Battery Reaction Time (CANTAB eclipse 5.0; Cambridge Cognition Ltd. http://www.camcog.com) touchscreen version (MT = movement time for correct responses; RT = mean simple reaction time for correct responses); Pegboard = Purdue pegboard task; § PSQI = Pittsburgh Sleep Quality Index. Sub-item 11c: “How often in the past month have you had legs twitching or jerking while you sleep?” 0 = not during the past month, 1 = less than once a week, 2 = once or twice a week, 3 = three or more times a week

**Table 2:** Details of variables and tests

Domain	Explanatory variables of interest^a^ (predictors)	Analysis performed	N (subjects with available data)
Demographics	Age, sex, handedness	Linear regression	482
Impulsive behavior	Alcohol consumption, current smoking, Body Mass Index (BMI)	Linear regression	477
Self-paced tasks	Verbal fluency, semantic fluency	Linear regression	486
Mood	CES-D	Partial correlation	485
Motor performance and sleep	CANTAB RTI (MT, RT), Pegboard (left, right, both, assembly), PSQI_11c	Linear regression	270

Legend: CES-D = Centre for Epidemiological Studies Depression Scale; CANTAB RTI = Cambridge Neuropsychological Test Automated Battery Reaction Time (CANTAB eclipse 5.0; Cambridge Cognition Ltd. http://www.camcog.com) touchscreen version (RT = mean simple reaction time for correct responses, MT = movement time for correct responses); Pegboard = Purdue pegboard task; PSQI = Pittsburgh Sleep Quality Index

^a^Volume of BG structures was included as covariate in all (ROI and voxel-wise) analyses

For the ROI analysis, the average PE of the BGN was extracted for each subject within a mask including caudate nucleus, putamen, and globus pallidus, from the Harvard-Oxford probabilistic atlas (threshold 30%) as a measure of FC. The analyses were then performed with SPSS (version 24.0) to evaluate the variance in FC explained by the variables explored in our study. All independent variables entered into the regression models at the same time (enter method or forced entry). Further analyses were performed on the significant predictors on the single BG structures (with Bonferroni correction across structures).

Whole-brain voxel-wise analyses of the BGN maps were performed to investigate the possible relationships between the explanatory variables and FC of the BGN with no restriction to the basal ganglia, to allow exploring possible associations also in cortical areas. Significance of associations were tested using a non-parametric permutation test (randomise, part of FSL (Winkler et al. 2014)) and results were considered significant for p < 0.05 after correction for multiple comparisons using the threshold-free cluster enhancement (TFCE) approach.

## Results

**Fig. 1:**
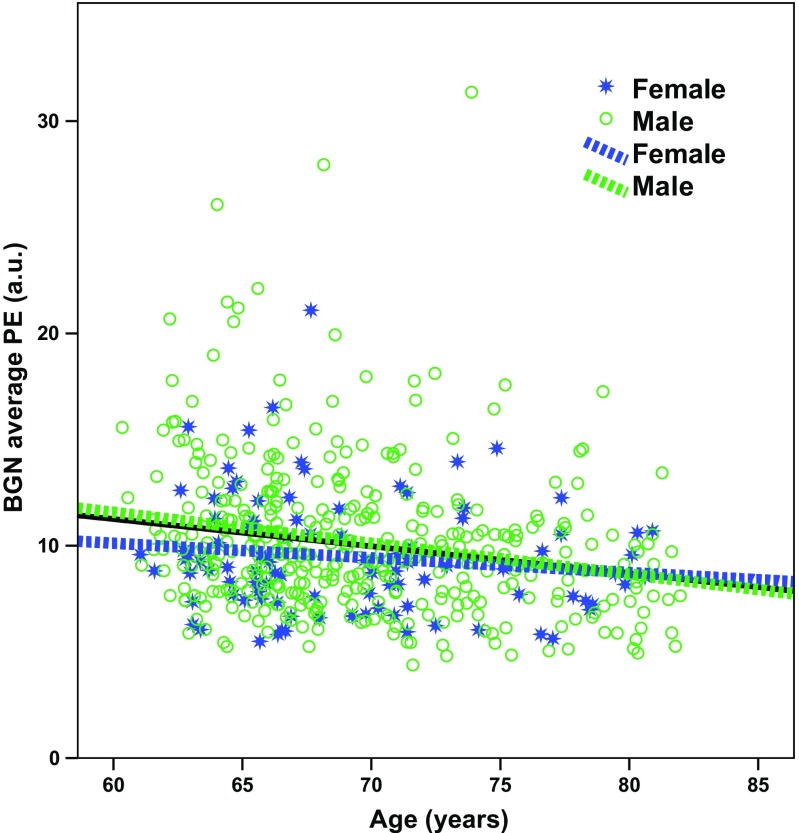
**ROI analysis results**. Statistically significant negative correlation was found between PE values extracted from the whole BGN and age (black solid line shows the linear fit across all subjects). The negative correlation between FC and age was non-significantly stronger in males (green) than females (blue)

In the ROI analysis, the only analysis that led to significant results was the multiple linear regression that included demographic variables. The model explained 4.8% of the variance in the data when looking at all the structures together (adjusted R^2^ = 0.48, p < 0.001). Age (beta = − 0.212, p < 0.001) and sex (M > F, beta = 0.117, p = 0.014) were significant predictors. Figure 1 illustrates the negative correlation between FC connectivity in the BGN and age. Further investigation of correlations between FC within the single structures and age in the full sample showed significant negative correlation (p < 0.01, Bonferroni corrected across six structures) between age and bilateral caudate (Spearmann’s rho left = − 0.256, right = − 0.232) and bilateral putamen (Spearmann’s rho left = − 0.182, right = − 0.187). No significant differences (Bonferroni corrected across structures) between men and women were observed (all p > 0.05). When splitting the subjects by gender, the negative correlation between FC and age remained significant for men (Spearmann’s rho = − 0.228, p < 0.001), but not for women (Spearmann’s rho = − 0.149, p = 0.144) (Fig. 1). However, the difference between the two correlation coefficients tested using Fisher r-to-z transformation (Myers and Sirois 2006) was not statistically significant (z = − 0.72, p = 0.472).

**Fig. 2:**
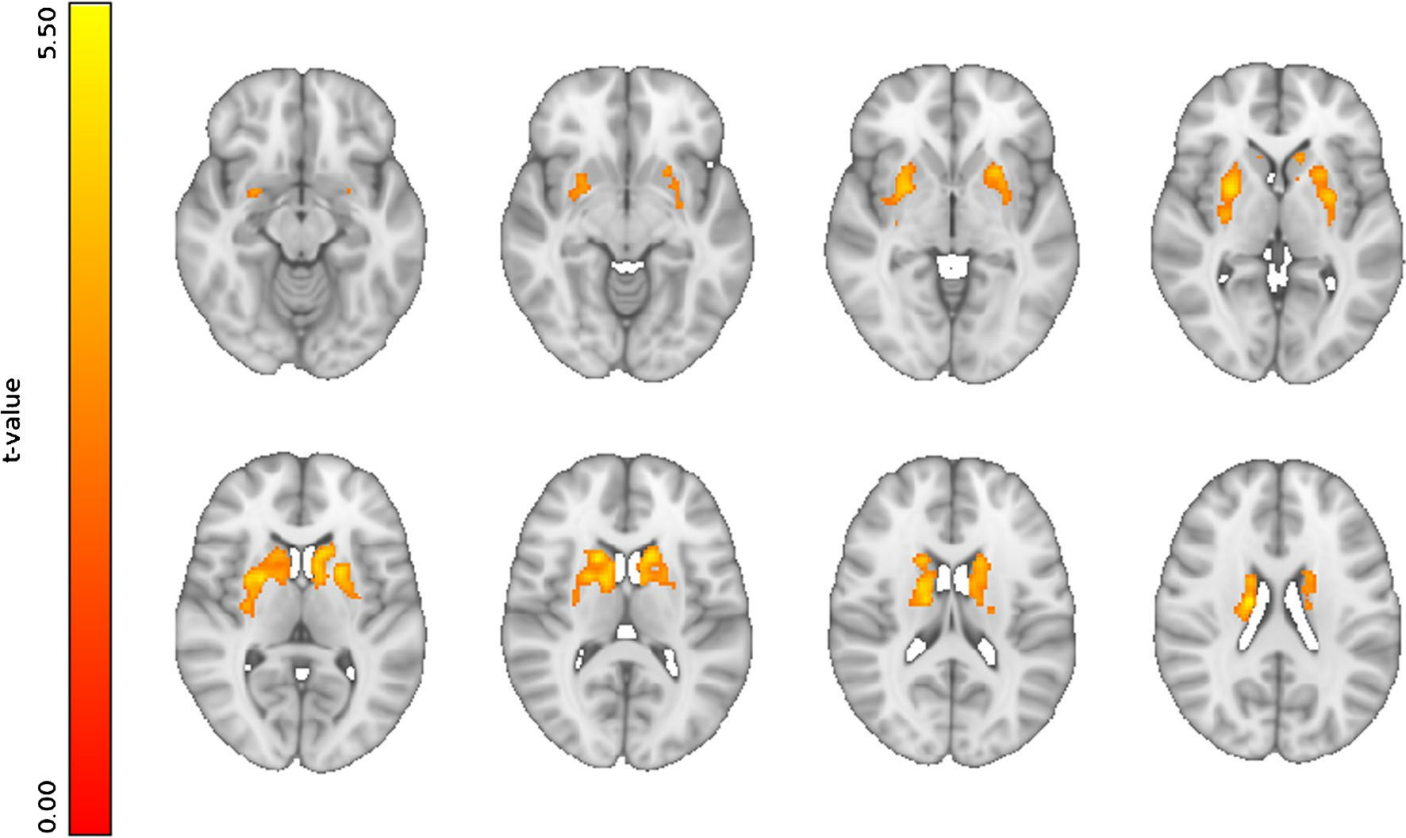
**Voxel-wise results**. Significant negative correlation (p_TFCE_<0.05) was found between FC in the BGN and age. Images are shown in radiological orientation. (all statistical maps are available at: https://neurovault.org/collections/2681)

Similar to the ROI analyses, the voxel-wise analyses showed significant results only when testing demographic variables. Age, the only significant correlate, showed a significant negative correlation with FC in the BGN in the bilateral putamen (clusters’ peak MNI coordinates (x,y,z): left=(− 24,12,− 4); right=(28,6,− 6)), extending into bilateral caudate nucleus, bilateral thalamus, and bilateral amygdala (Fig. 2). No other significant associations were found with the remaining variables/domains (all statistical maps are available at: https://neurovault.org/collections/2681).

## Discussion

The aim of this exploratory study was to better understand the variability of functional connectivity within the basal ganglia resting state network in healthy older adults, with particular focus on potential links to dopamine-related function. We investigated the association between FC and variables related to demographics, impulsive behavior, self-paced tasks, mood, and motor variables in a large cohort of 486 participants in the Whitehall-II imaging sub-study. We found that age was the strongest correlate of FC in the BGN. In particular, a significant negative correlation between age and FC was observed in several basal ganglia structures in both the ROI and voxel-wise analyses.

It is known that aging affects FC in general (Biswal et al. 2010) and that FC in the default mode network decreases in older age (Andrews-Hanna et al. 2007; Damoiseaux et al. 2008). Regarding the BGN, the relationship between FC and age described by (Sole-Padulles et al. 2016) and (Allen et al. 2011) was positive rather than negative. However, the age ranges in these two studies (7–18 and 12–71 years respectively) were very different from ours, which focused on elderly subjects (60–82 years). In this framework, our results add complementary information to the current literature, suggesting an inverted U-shaped pattern of FC of the BGN across the life span.

The effect of age on the BG has been previously shown with PET and SPECT. The review by (Reeves et al. 2002) showed an association between age and loss of striatal dopamine transporters (DATs) in the caudate and putamen in both hemispheres (Wong et al. 1984; van Dyck et al. 2002). Those findings suggest that the changes underlying the negative effect of aging on the FC within the BGN may be related to biological alterations of the dopaminergic system, like those involved in PD. In fact, previous studies using SPECT (Ba and Martin 2015) and rfMRI (Szewczyk-Krolikowski et al. 2014; Rolinski et al. 2015) found differences in idiopathic PD compared to controls within the BG in terms of DATs and FC. These areas are the same that show a negative correlation with age in our sample of healthy aging participants.

It needs to be acknowledged that the subjects are from a cohort of civil servants recruited in 1985 (Marmot and Brunner 2005) and therefore not entirely representative of the general population. In particular, the observed correlation with age that seems to be driven by males, would need further investigation on a more balanced sample, due to the gender bias in this cohort.

Regarding the relationship between FC and other dopamine-related behavioral data, none of the tested domains showed significant correlations with FC in the BGN. This could be due to multiple reasons: on one hand, the variables we tested were selected because they can be influenced by dopamine, but, since they reflect also other functions, may not be strictly related to the dopaminergic changes in the BG. On the other hand, the variability we observed in FC in the BG is possibly a sum of dopamine-related and dopamine-unrelated processes and therefore not strictly related to the dopamine-related behavioral data. Aging is a process that incorporates multiple domains, including dopamine-related changes, and might better explain the FC variability observed in the BGN with rfMRI.

In light of these results, our findings might have implications for the development of imaging biomarkers, for example for the detection of PD, which must have age-norms to be maximally useful. In fact, since age accounts for some of the spread in FC of healthy subjects, statistically controlling for its effect might increase the specificity of a biomarker based on BGN functional connectivity.
